# Role of error-prone DNA polymerases in spontaneous mutagenesis in *Caulobacter crescentus*

**DOI:** 10.1590/1678-4685-GMB-2018-0283

**Published:** 2020-03-09

**Authors:** Alexy O. Valencia, Vânia S. Braz, Magna Magalhães, Rodrigo S. Galhardo

**Affiliations:** 1 Universidade de São Paulo, Instituto de Ciências Biomédicas, Departamento de Microbiologia, São Paulo, SP, Brazil.

**Keywords:** DinB, ImuC, DnaE2, spontaneous mutagenesis, Caulobacter crescentus

## Abstract

Spontaneous mutations are important players in evolution. Nevertheless, there is a paucity of information about the mutagenic processes operating in most bacterial species. In this work, we implemented two forward mutational markers for studies in *Caulobacter crescentus*. We confirmed previous results in which A:T → G:C transitions are the most prevalent type of spontaneous base substitutions in this organism, although there is considerable deviation from this trend in one of the loci analyzed. We also investigated the role of *dinB* and *imuC*, encoding error-prone DNA polymerases, in spontaneous mutagenesis in this GC-rich organism. Both *dinB* and *imuC* mutant strains show comparable mutation rates to the parental strain. Nevertheless, both strains show differences in the base substitution patterns, and the *dinB* mutant strain shows a striking reduction in the number of spontaneous -1 deletions and an increase in C:G → T:A transitions in both assays.

## Introduction

Spontaneous mutations, arising without exposure of cells to external genotoxic agents, arise at a constant rate in all organisms (Drake, 1991; Lynch, 2010). Although most organisms show a bias towards C:G → T:A transitions among spontaneous base substitutions, we have recently found that the bacterium *C. crescentus* shows a different trend, with more A:T → G:C substitutions among spontaneous mutations in the *rpoB* gene (Martins-Pinheiro *et al.*, 2017). Nevertheless, use of *rpoB* as a mutational marker has limitations, such as the relatively small number of amino acid changes leading to the detectable phenotype (Rif^R^) and the lack of detection of insertions and deletions.

Error-prone polymerases are widespread in nature (Ohmori *et al.*, 2001), playing an important role in DNA damage tolerance in bacteria by promoting translesion DNA synthesis (TLS) (Fuchs and Fujii, 2013). As a direct consequence of their TLS activity and their regulation by many cellular stress responses, error-prone polymerases are likely important players in the mutational processes both in growing and non-growing bacterial cells (Galhardo *et al.*, 2007). These enzymes have been extensively studied in *Escherichia coli*, where the SOS-regulated genes *umuDC* and *dinB* encode the error prone polymerases Pol V and Pol IV respectively.

The role of these enzymes in DNA damage tolerance in *E. coli* is clear. Pol V is required for damage-induced mutagenesis after cellular exposure to a number of different DNA damaging agents, such as UV light, methyl nitrosoguanidine, and 4-NQO (Kato and Shinoura, 1977; Bagg *et al.*, 1981; Woodgate, 1992). Pol IV is involved in error-free bypass of both alkylation damage (Bjedov *et al.*, 2007) and N^2^-guanine adducts (Jarosz *et al.*, 2006). Deletion of the *dinB* gene does not affect the rate of spontaneous mutations (Mckenzie *et al.*, 2003; Kuban *et al.*, 2004), and neither the sequences of the mutations observed in the *rpoB* gene (Wolff *et al.*, 2004). Due to the very tight transcriptional and post-transcriptional control of Pol V activity (Goodman *et al.*, 2016), *umuDC* genes are assumed to have little effect on spontaneous mutagenesis. On the other hand, both DinB and UmuDC have been implicated in untargeted mutagenesis in SOS-constitutive cells (Caillet-Fauquet and Maenhaut-Michel, 1988; Kim *et al.*, 1997, 2001), and also in stress-induced mutagenesis (Cirz *et al.*, 2005; Petrosino *et al.*, 2009). Additionally, *dinB* is subject to many layers of regulation in *E. coli*, being induced by the SOS response (Kenyon and Walker, 1980), upon entry into stationary phase (Layton and Foster, 2003) and by beta-lactam antibiotics (Pérez-Capilla *et al.*, 2005).

*C. crescentus* bears two genes encoding error-prone polymerases in its genome, *dinB*, and *imuC* (*dnaE2*), the later one being part of a conserved operon also containing *imuA* and *imuB*. Previous studies have shown that this operon is part of the SOS response both in *C. crescentus* and in other bacteria where these three genes are induced as part of the SOS response and cooperate in a mutagenesis pathway responsible for Mitomycin C- and UV-induced mutagenesis (Boshoff *et al.*, 2003; Galhardo *et al.*, 2005; Warner *et al.*, 2010). Nevertheless, constitutive transcription of *imuABC* in SOS-induced levels does not promote significant increases mutation rates in *C. crescentus*, suggesting a tight control of this mutagenesis pathway in cells experiencing DNA damage (Alves *et al.*, 2017). Furthermore, the same study showed that the activity of ImuABC is RecA-independent, setting it apart from the paradigm of mutagenic DNA polymerase regulation in *E. coli*. Therefore, *imuABC* are thought of as functional substitutes of *umuDC* in bacteria lacking these genes, although its properties and regulation show considerable differences.

On the other hand, the physiological role of *dinB* in *C. crescentus* is still not understood. Differently from *E. coli*, this gene is not part of the SOS regulon (Galhardo *et al.*, 2005; da Rocha *et al.*, 2008), and is not upregulated in response to UV light, hydroxyurea and mitomycin C (Modell *et al.*, 2011).

In an attempt to better understand the physiological role of error-prone polymerases and to obtain a better appraisal of the characteristics of spontaneous mutagenesis in *C. crescentus*, we implemented two forward mutational assays. With these tools, we investigated the characteristics of spontaneous mutagenesis in *C. crescentus* and analyzed the role of DinB and ImuC in this process. We found that DinB has a major role in the generation of spontaneous deletions in the *C. crescentus* genome.

## Materials and Methods

### Bacterial strains and growth conditions

The bacterial strains and plasmids used in this work are listed in Table 1. *C. crescentus* strains were grown in PYE or M2 glucose (Ely, 1991), at 30 ^o^C with constant shaking at 250 rpm for liquid cultures. When needed, the following concentrations of antibiotics were used: ampicillin 200 μg/mL (for selection of Amp^R^ mutants in the *xylbla* assay); tetracycline 4 μg/mL (for selection of Tet^R^ mutants in the *cItet* assay); kanamycin 5 μg/mL; nalidixic acid 20 μg/mL.

**Table 1:** Bacterial strains and plasmids used in this study.

Strain	Relevant Genotype	Source
*C. crescentus*		
NA1000	Parental strain, *C. crescentus* CB15 derivative	Evinger and Agabian, 1977
CS606	NA1000 Δ*blaA*	West *et al.,* 2002
GM40	NA1000 *imuC*::Spec^R^	Galhardo *et al.,* 2005
GM50	NA1000 *dinB*::Spec^R^	Galhardo *et al.,* 2005
RSG113	NA1000 Δ*blaA* Δ*xylX*::*blaA*	This study
RSG124	NA1000 Δ*blaA* Δ*xylX*::*blaA dinB*::Spec^R^	This study
RSG247	NA1000 Δ*blaA* Δ*xylX*::*blaA imuC*::Spec^R^	This study
RSG317	NA1000 *cI* (Ind^-^) λpR *tetA*	This study
RSG318	NA1000 *cI* (Ind^-^) λpR *tetA imuC*::Spec^R^	This study
RSG319	NA1000 *cI* (Ind^-^) λpR *tetA dinB*::Spec^R^	This study
*E. coli*		
MG1655, *cI* marker	MG1655 attλ:*cI* (Ind^-^) λpR *tetA* Δ*ara*:FRT Δ*metRE*:FRT	Bjedov *et al.,* 2007)
Plasmids		
pNPTS138	pNPTS129 derivative, *oriT sacB* Kan^R^	Tsai and Alley, 2001
pNPTxylblaE2	In frame substitution of *xylX* by *blaA* with flanking regions, cloned in pNPTS138	This study
pMCS7	Cloning vector, non-replicating in *C. crescentus*	Thanbichler *et al.,* 2007
pMCSCI	pMCS7 containing the *cItet* cassette and ~500 bp of DNA for homologous recombination in the *C. crescentus* chromosome	This study

### Introduction of the *cI* (Ind^-^) λpR *tetA* marker in the *C. crescentus* genome

The *cI* (Ind^-^) λpR *tetA* cassette, hereafter referred to as *cItet* marker for simplicity, was originally constructed for use as a forward mutational marker in *E. coli* (Bjedov *et al.*, 2007). This marker scores mutations in the *cI* repressor gene, leading to constitutive expression of *tetA*, and therefore, tetracycline resistance. For integration of this marker in the *C. crescentus* chromosome, a 538 bp fragment corresponding to the region between bases 2404103 and 2404622 of the NA1000 genome was amplified using primers inter3fwd and inter3rev (Table S1). This fragment served as the homology region for recombination of the final construct on the chromosome. This amplicon was cloned in the pMCS7 integrative vector in the *Nde*I site, using restriction sites introduced in the primers. The resulting plasmid was digested with *Kpn*I and *Sma*I to receive the *cI* cassette. The *cI* cassette was amplified from the genomic DNA of the MG1655 *cI* (Ind^-^) λpR *tetA E. coli* strain using the cItetfowkpn and cItetrevsac primers (Table S1) and cloned in the above construct. The resulting plasmid, pMCSCI (Gent^R^), was introduced in *E. coli* S17.1 by electroporation, and passed to *C. crescentus* NA1000 via conjugation, resulting in strain RSG317, which yielded spontaneous Tet^R^ mutants, unlike the parental strain NA1000. *dinB* and *imuC* derivatives of RSG317 were constructed using ΦCr30 transduction using GM40 and GM50 strains as donors.

### Construction of the *xylbla* marker

**Figure 1:**
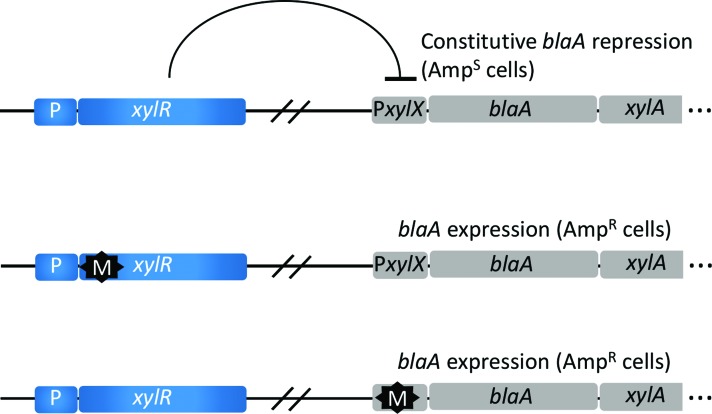
Rationale of the xylbla marker. The xylX gene in the xyl operon has been replaced by blaA. Nevertheless, repression by XylR in the absence of xylose renders cells phenotypically AmpS. Cells can become phenotypically AmpR by loss of function mutations (M) in xylR, or by mutations in the xylR operator inside PxylX.

We envisaged a strategy to replace the *xylX* gene, which is necessary for the metabolism of xylose but dispensable for growth in rich media (Stephens *et al.*, 2007a), by *blaA*, conferring resistance to ampicillin (West *et al.*, 2002), to construct a novel marker for mutagenesis studies using a native *C. crescentus* gene. The resulting strain is phenotypically Amp^S^ in the absence of xylose, but Amp^R^ after mutations (i) inactivating the XylR repressor, and (ii) altering the XylR operator sequences in the P*xylX* promoter (Figure 1). This marker is referred to throughout the text as *xylbla*.

To replace *xylX* by *blaA*, we constructed plasmid pxylblaE2, containing *blaA* flanked by 5` and 3` homology regions surrounding *xylX*, as follows. First, blaA and blaB primers were used for the amplification of *blaA* flanked by *Eco*RI and *Bam*HI restriction sites. A region of homology immediately 3`of *xylX* was amplified using primers xylC and xylD, which introduced *Bam*HI and *Spe*I restriction sites 5 and 3` respectively. A large fragment containing the whole *xylX* gene and flanking regions was obtained with primers xylA (introducing a *Hind*III site in the 5`portion) and xylD. This amplicon possesses a natural *Eco*RI site. Digestion of this fragment with *Hind*III and *Eco*RI produced a shorter fragment of 636 bp, corresponding to the region immediately 5` to *xylX* plus the first 18 bp of the open reading frame. Ligation of the three fragments in pBC KS + yielded a construct containing *blaA* flanked by regions of homology to the 5` and 3` of *xylX*. This fragment was subcloned in pNPTS138 to produce the pNPTxylblaE2 construct.

The first recombination event, integrating pNPTxylblaE2 into the *C. crescentus* chromosome, was obtained by conjugation of *C. crescentus* CS606 (Δ*blaA*) with *E. coli* S17.1 carrying pNPTxylblaE2, selecting for Kan^R^ Nal^R^ conjugants. Afterwards, a second recombination event, leading to plasmid loss, was selected by plating cells in PYE medium supplemented with 3% sucrose. The resulting clones were screened for xylose auxotrophy, to look for strains with the substitution of *xylX* by *blaA*. As expected, these recombinants yielded spontaneous Amp^R^ mutants, unlike the parental strain CS606. One of these recombinants, designated RSG113, was chosen for the experiments. *dinB* and *imuC* derivatives of RSG113 were constructed using ΦCr30 transduction, using GM40 and GM50 strains as donors.

### Fluctuation tests and determination of mutation sequences

Fluctuation tests for measurement of mutation rates were initiated by diluting a saturated culture to ~10^2^ cells/mL in PYE medium. This diluted culture was split in 11 tubes containing 1 mL of cell suspension each, which were grown for 48 h at 30 ^o^C. Cell viability was determined by serial dilution and plating on PYE. The number of mutants was determined as follows for each of the markers used for mutagenesis studies. Due to the high frequency of mutants, in the assays using the *xylbla* and *cItet* markers, 100 μL of each culture was plated in duplicate in PYE Amp and PYE Tet respectively. Mutation rates were calculated by the Ma-Sandri-Sarkar Maximum Likelihood Estimator (MSS-MLE) using the FluCalc web tool (Radchenko *et al.*, 2018).

All the Tet^R^ and Amp^R^ mutants sequenced for the determination of the mutational spectrum come from independent cultures in the fluctuation assays, to ensure independent mutations were assessed. Mutations were detected and analyzed for sequence quality using the Genious R8 software (Biomatters).

To determine the sequences of the Tet^R^ mutations, the *cI* gene was amplified using primers cItetfwd and cItetrev. Purified PCR products were sequenced using primers cIfwd, cIrev, cItet-Seq and cItet-int.

Since two classes of mutants are detectable in the *xylbla* assay, we first identified the two classes of mutants using a previously reported inability of *xylR* mutants to grow on minimal medium containing glucose as a carbon source. Therefore, Amp^R^ colonies were first spotted on PYE and M2 glucose media. Mutants unable to grow on minimal media were sequenced for mutations in *xylR*, and the remainder were sequenced for mutations on P*xylX*. To sequence *xylR*, a PCR product was obtained with primers xylR-fwd and xylR-rev, and subsequently sequenced with the same primers and xylR-seq-final, xylR-seq-1. Mutations in P*xylX* were determined by PCR with primers Pxylx-fwd and blaB, and sequenced with Pxylx-fwd and PxylX-seq.

## Results

### Forward mutational assays in *C. crescentus*

In order to have better experimental tools for the study of mutagenesis in *C. crescentus*, we constructed two sets of strains (parental, and its *dinB* and *imuC* derivatives) containing different forward mutational assays. The first set contains the *cItet* marker, previously developed for mutagenesis studies in *E. coli* (Bjedov *et al.*, 2007). The other contains a newly developed marker, *xylbla* (Figure 1). It uses the well-known xylose inducible *xylX* promoter, which is controlled by the LacI family repressor XylR (Meisenzahl *et al.*, 1997; Stephens *et al.*, 2007b). In this system, the *bla* gene encoding a beta-lactamase naturally present in *C. crescentus* was put under control of P*xylX*, rendering cells phenotypically Amp^S^. Two types of mutations are conceivable in this system. Mutations that disrupt the operator sequence in P*xylX*, and mutations that inactivate the XylR repressor, as depicted in Figure 1. *xylR* and P*xylX* mutants can be distinguished based on the poor growth of the former on minimal media containing glucose as the sole carbon source, as reported before (Stephens *et al.*, 2007b). *C. crescentus* cells carrying *cItet* give rise to spontaneous Tet^R^ mutants, and those carrying the *xylbla* marker give rise to spontaneous Amp^R^ mutants, unlike the NA1000 strain. Sequencing revealed that both Amp^R^ and Tet^R^ mutants carry mutations in the predicted targets (see below). Therefore, we successfully used forward mutational assays for this model organism.

### Roles of *dinB* and *imuC* in spontaneous mutagenesis

**Figure 2:**
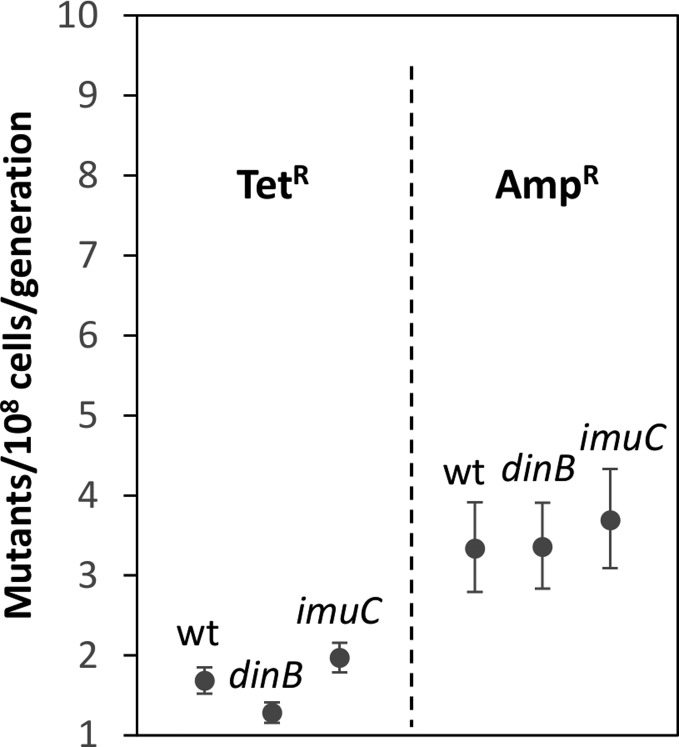
TetR and AmpR mutation rates. TetR mutation rates were determined using 66 cultures from 6 independent experiments. AmpR mutation rates were determined using 55 cultures from 5 independent experiments. Both the parental strains containing the cItet and xylbla markers (wt) and their dinB and imuC derivatives were analyzed. Mutation rates and 95% confidence intervals (represented by the error bars) were calculated using the MSS-MLE (Ma-Sandri-Sarkar Maximum Likelihood Estimator).

We sought to determine the role of *dinB* and *imuC* in spontaneous mutagenesis using both markers in fluctuation assays (Figure 2). The results show that both the *dinB* and *imuC* strains show Amp^R^ and Tet^R^ mutation rates comparable to the respective parental strains. In the case of *imuC*, mutation rates are indistinguishable from the parental strains ones in both markers, given the overlap in the confidence intervals. The *dinB* strain shows comparable Amp^R^ mutation rates, but slightly decreased Tet^R^ mutagenesis. Nevertheless, the small difference observed (less than 2-fold) is usually not considered biologically relevant. These results confirm our previous observations using the more limited *rpoB* marker, which can only detect base substitutions. In those experiments, we showed that *imuC* does not influence the rate of Rif^R^ mutations (Martins-Pinheiro *et al.*, 2017). Although these results indicate that both DinB and ImuABC have a limited role in spontaneous mutagenesis, we reasoned that quantitative determination of mutation rates lack the sensitivity to detect small, but biologically important, changes in the mutational signatures in cells lacking these polymerases, as exemplified by the small differences in Tet^R^ mutants observed in the *dinB* strain. Therefore, we proceeded to analyze the sequences of spontaneous mutations found in all loci under study.

### Spontaneous mutation signatures in *cI*

**Figure 3:**
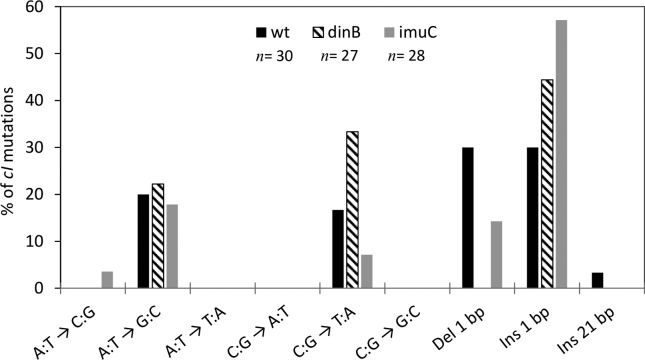
Distribution of the different base substitutions in cI in wt, dinB and imuC strains. Results are shown for NA1000 strain (wt) and mutant strains (dinB and imuC). n indicates the number of mutants analyzed in each strain. The different base substitutions are indicated. Del 1 bp: 1 bp deletions. Ins 1 bp: 1 bp insertions. Ins 21 bp: 21 bp insertion detected in the wt strain.

We analyzed the sequences of Tet^R^ mutants obtained with the set of strains containing the *cItet* marker, and the results are represented in Figure 3. The positions of all mutations analyzed are described in Table S2. In all strains, small indels account for a large fraction of the mutations observed, in agreement with studies in other organisms using similar markers (Schaaper *et al.*, 1986). All these mutations are localized in homopolymeric runs in *cI* (data not shown). Both *dinB* and *imuC* strains show alterations in the number of such frameshifts. 1 bp deletions represent 30% of the mutations observed in the parental strain, and approximately 15% of the mutations observed in the *imuC* derivative. No -1 frameshifts were detected in the *dinB* strain among the Tet^R^ mutants analyzed. On the other hand, both *dinB* and *imuC* deficiencies lead to an increase in the number of 1 bp insertions in *cI*.

Previously, we have described that the spontaneous base substitution signature of *C. crescentus* in the *rpoB* gene is dominated by A:T → G:C transitions, which is different from the bias towards C:G → T:A observed in most organisms studied to date (Martins-Pinheiro *et al.*, 2017). The sequences of mutations in *cI* confirm this trend, given that A:T → G:C changes outnumber C:G → T:A transitions in the wt background. Curiously, the number of C:G → T:A substitutions is increased in the *dinB* background, but decreased in *imuC*.

### Spontaneous mutation signatures in *xylR*

**Figure 4:**
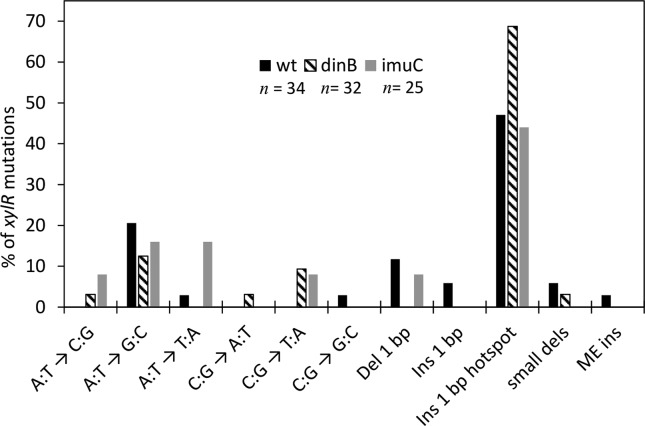
Distribution of the different base substitutions in xylR in wt, dinB and imuC strains. The different base substitutions are indicated. Del 1 bp: 1 bp deletions. Ins 1 bp: 1 bp insertions not located in the hotspot. Ins 1 bp hotspot: 1 bp insertions located in the hotspot. Small dels: 2-8 bp deletions. ME ins: insertion of mobile elements. n indicates the number of mutants analyzed in each strain.

The spectrum of spontaneous mutations in *xylR* is summarized in Figure 4. The positions of all mutations analyzed are described in Table S3. This gene has a very pronounced mutational hotspot, in which a cytosine insertion occurs after base 230 of the open reading frame (Figure S1). Interestingly, this hotspot does not consist of a homopolymeric run, and does not present any obvious secondary structure formation. Therefore, the basis for the presence of this hotspot is not known. The *dinB* strain shows a marked increase in the proportion of mutations in this hotspot.

Apart from mutations in the hotspot, the *dinB* strain lacks 1 bp deletions in homopolymeric runs in *xylR*, as seen in *cI*, suggesting that those are, to a large extent, generated by Pol IV activity in *C. crescentus*. Another feature in common between the two markers is that A:T → G:C transitions are the most frequent type of base substitution observed in the wt strain. The same is observed for *dinB* and *imuC* mutants in *xylR*. Both *dinB* and *imuC* deficiency cause an increase in the number of C:G → T:A mutations in *xylR*, a feature not observed in the *cI* gene for the *imu*C mutant, in which we observed the opposite effect. Nevertheless, *din*B deficiency leads to an increase of C:G → T:A in both markers. Other differences in the patterns of base substitutions in *xylR* can be seen among the strain backgrounds, with the *imuC* strain showing more A:T → T:A and A:T → C:G transversions. Taken together, the results obtained with the two loci point to a clear role of *dinB* in preventing 1 bp insertions and C:G → T:A transitions, and in the formation of -1 bp frameshifts. Minor changes in the mutational spectrum can be seen in the *imuC* strain. This polymerase seems to have a role in preventing A:T → T:A mutations in *xylR* and A:T → C:G transversions in both markers.

### Spontaneous mutation signatures in P*xylX*

**Figure 5:**
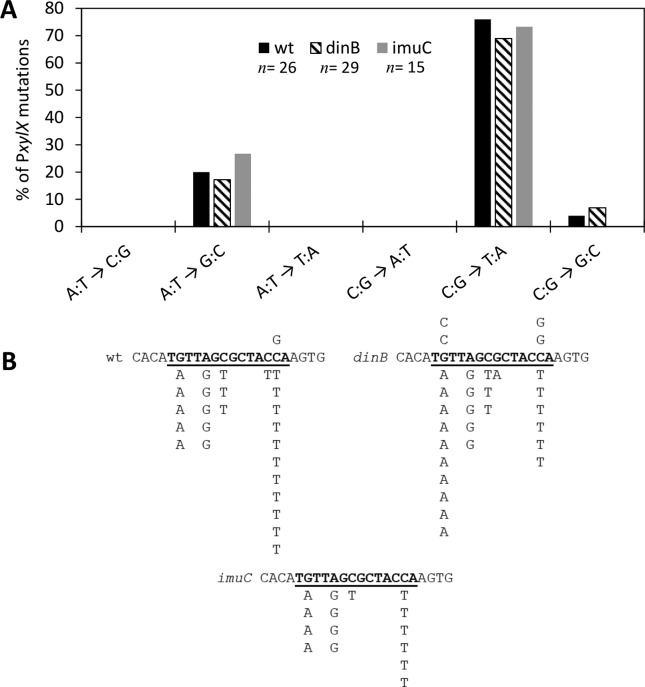
PxylX mutations. (A) Distribution of the different base substitutions in PxylX in wt (parental strain), dinB and imuC strains. The different base substitutions are indicated. n indicates the number of mutants analyzed in each strain. (B) A small region in the PxylX region is shown, with the XylR binding site underlined. Sequences above and below the line show the different mutations detected.

We also investigated the Amp^R^ mutations localized in P*xylX* in cells carrying the *xylbla* marker. Although the 14 bp operator sequence is a very small mutational target compared to the 1.25 kp long *xylR* ORF, we observed that P*xylX* mutations correspond to approximately 1/4 of all Amp^R^ mutations in cells carrying *xylbla* (data not shown). There is no significant variation among the three strain backgrounds, but there is a remarkable reversion of the mutational bias observed in *rpoB* (Martins-Pinheiro *et al.*, 2017), *cI* and *xylR* (Figure 5A). Here, we find that C:G → T:A mutations largely outnumber A:T → G:C transitions. C:G → T:A mutations are detected in 3 independent positions within the XylR operator located in P*xylX*, ruling out a hotspot to account for the results (Figure 5B). Another striking point is that no frameshifts were observed among all the P*xylX* mutations analyzed.

## Discussion

In this work, we have successfully implemented two forward markers for mutagenesis studies in *C. crescentus*. Using these tools, we confirmed previous observations in which a A:T → G:C mutations are the most prevalent type of base substitution observed in *C. crescentus* (Martins-Pinheiro *et al.*, 2017). Curiously, this is the predominant type of mutation found in mismatch repair deficient *E. coli*, but not wild type cells (Lee *et al.*, 2012). In wild type *E. coli*, as well as in many other organisms, C:G →A:T mutations predominate (Lee *et al.*, 2012), and have been proposed as the universal mutational bias in bacteria (Hershberg and Petrov, 2010; Hildebrand *et al.*, 2010). These forward mutational markers will be valuable tools for future studies aiming at understanding such difference between *C. crescentus* and most other organisms.

Nevertheless, we spotted an interesting deviation of this A:T → G:C bias in *C. crescentus*. When mutations in the P*xylX* region werere analyzed, there was a clear shift in the predominant mutation type, with C:G → T:A transitions being the most frequent base substitution. The basis for this deviation is not yet understood. We envision two not mutually exclusive explanations. First, it is possible that lesions giving rise to C:G →T:A transitions, such as uracil residues formed by spontaneous cytosine deamination, are repaired more efficiently in transcribed regions compared to non-transcribed ones. Second, the constant binding of the XylR repressor to the operator sequence could hinder the access of repair proteins to DNA lesions and/or affect the rate of lesion formation. These two hypotheses could also help to explain the proportionally higher mutation rates in the small P*xylX* target.

We also analyzed the role of the error-prone DNA polymerases ImuC and DinB in spontaneous mutagenesis. *imuC* is controlled by the SOS response in *C. crescentus*, whereas *dinB* is not (Galhardo *et al.*, 2005; da Rocha *et al.*, 2008). Additionally, no conditions where *dinB* expression is increased has been found in high throughput studies under DNA damaging conditions (Modell *et al.*, 2011). Therefore, to the best of our knowledge, *dinB* expression is constitutive in *C. crescentus*, unlike in other bacteria, such as *E. coli* and *Pseudomonas aeruginosa* (Courcelle *et al.*, 2001; Sanders *et al.*, 2006), but similar to *M. tuberculosis,* where the two *dinB* orthologs are not part of the SOS regulon (Kana *et al.*, 2010; Smollett *et al.*, 2012). This observation is reminiscent of the data in *C. crescentus* and indicates that inducibility by DNA damage is not a universal feature of *dinB* in bacteria. Furthermore, the *M. tuberculosis* orthologs have no obvious role in DNA damage tolerance, and do not influence the rate and spectrum of spontaneous mutagenesis (Kana *et al.*, 2010).

In *C. crescentus* we found that this polymerase plays a role in spontaneous mutagenesis, given that 1 bp deletions seem to be totally DinB-dependent. Interestingly, ImuC also plays a role in the genesis of this same type of mutation. These mutations typically arise in homopolymeric runs, as a consequence of replication slippage. DinB overexpression has been long known to lead to an increase in the number of 1bp deletions (Kim *et al.*, 1997, 2001), which occur through a dNTP-stabilized misalignment (Kobayashi *et al.*, 2002). Nevertheless, our data suggest that physiological levels of DinB promote such mutagenesis in *C. crescentus*. Future studies are needed to understand if this phenomenon happens during TLS of endogenous lesions, or simply by gaining access to ongoing replication of undamaged templates. Another possibility is that DinB may be mutagenic in DNA synthesis during recombination intermediates (Pomerantz *et al.*, 2013). Stress-induced mutagenesis in non-growing cells also has a strong *dinB*-dependence (Mckenzie *et al.*, 2001). It could be the case that upon saturation of the cultures and cessation of growth, a *dinB*-dependent stress-induced mechanism is triggered in *C. crescentus*, contributing to the appearance of the DinB-dependent frameshifts in the fluctuation assays. Nevertheless, cultures were plated only a few hours after they reached saturation in our experimental conditions, and not after the longer periods of time required to detect stress-induced mutations (Shee *et al.*, 2011).

Also, in both *cI* and *xylR* the absence of *dinB* leads to an increase in the proportion of C:G →T:A transitions. This may indicate a role of this constitutively expressed polymerase in maintaining the A:T →C:G bias in *C. crescentus*. Other types of mutations were found to be influenced by error-prone polymerases, such as the A:T → C:G and A:T → T:A transversions in *xylR*, both increased in the *imuC* background. In *cI*, ImuC seems to contribute to the formation of C:G → T:A transitions. The loci specificity of these observations probably reflects local sequence contexts that may either favor increased endogenous lesion formation, or hinder DNA repair, providing lesion substrates for translesion synthesis by these polymerases. This is evident in the case of *xylR* mutations in the *imuC* strain, where some of the A:T → T:A events occurred in the same position (Table S3).

Altogether, our results point to a role of DinB in the genesis of small deletions in *C. crescentus* cells not exposed to DNA-damaging agents. This study also demonstrates the importance of detailed analysis of mutational spectra, showing that it can reveal important small-scale changes in the proportion of base substitutions across different genetic backgrounds, which cannot be assessed by mere quantification of mutation rates.

## Supplementary material

The following online material is available for this article:

Figure S1 - Mutational hotspot in the *xylR* gene.

Table S1 - Primers used in this study.

Table S2 - Location of the mutations in the *cI* gene.

Table S3 - Location of the mutations in the *xylR* gene.
