# Supplementary material for: Role of error-prone DNA polymerases in spontaneous mutagenesis in Caulobacter crescentus
Source: Genet Mol Biol. 2020 Mar 9;43(1):e20180283. doi: 10.1590/1678-4685-GMB-2018-0283 (PMC7198004; doi:10.1590/1678-4685-GMB-2018-0283)
Supplement: Supplementary file 1 [file 1415-4757-GMB-43-1-e20180283-s2.pdf]

Supplementary Material to "Role of error-prone DNA polymerases in spontaneous mutagenesis in *Caulobacter crescentus*"

Table S1 - Primers used in this study.

**Primer**	**Sequence (5´ - 3´)**
blaA	GATGAATTCAAGCGCCTGATCCTGGCC
blaB	TCGCTTGGGCCCCGCCGCCT
cIfwd	TCAGCCAAACGTCTCTTCAG
cIrev	TACCAATGCGATCTTTGTCG
cItetFwd	ACTGGGCCCTCAGCCAAACGTCTCTTCAG
cItetfwdkpn	ACTGGTACCTCAGCCAAACGTCTCTTCAG
cItet-int	AAGCTTTCCTGACGGAATGT
cItetRev	GATGTCGACATTTGGTGACGAAATAACTAAG
Citetrevsac	GATGAGCTCATTTGGTGACGAAATAACTAAG
cItet-Seq	ATGAGCACAAAAAAGAAACC
inter3fwd	CATATGGCATCGATATCCGTGACTGG
inter3rev	CATATGCGCTGGCTATACCAAGGAGT
Pxylxfwd	GTCGGGCAGCAGGTACAAG
Pxylx-seq	CCTGATCCTCGCCCGAAA
xylA	GTCAAGCTTAGGAACCGTCCCTTCCAG
xylC	TACGGATCCTAATCGAAAGCTTCCCGC
xylD	CCTACTAGTCGTGATCAGGCCATAGACG
XylRfwd	TACCCGAAGCGGTCTAGTGT
XylRrev	TTGTCCGGAAAGAACGTACC
XylR-seq-1	AAGTGCGCCGAGCCGGAGCT
XylR-seq-final	TCACCCTCCCACCGTTC
