# Supplementary material for: Role of error-prone DNA polymerases in spontaneous mutagenesis in Caulobacter crescentus
Source: Genet Mol Biol. 2020 Mar 9;43(1):e20180283. doi: 10.1590/1678-4685-GMB-2018-0283 (PMC7198004; doi:10.1590/1678-4685-GMB-2018-0283)
Supplement: Supplementary file 3 [file 1415-4757-GMB-43-1-e20180283-s4.pdf]

Supplementary Material to "Role of error-prone DNA polymerases in spontaneous mutagenesis in *Caulobacter crescentus*"

Table S3 - Location of the mutations in the *xylR* gene**.** Position of each mutation is indicated, and the number of times each one was found is shown in parenthesis. For insertions and deletions in homopolymeric runs, the first position of the run is indicated. ND, not detected. ME, mobile element.

**Mutation type**	**wt**	***dinB***	***imuC***
Hotspot (ins 1 bp)	230 (16X)	230 (22X)	230 (11X)
Del 1 pb	75 (2X)<br>181 (1X)<br>567 (1X)	ND	75 (1X)<br>356 (1X)
Small dels	466-468 (1X)<br>724-741 (1X)	197-204 (1X)	ND
Ins 1 pb	99 (1X)<br>814 (1X)	ND	ND
A:T → G:C	-8 (3X)<br>146 (2X)<br>275 (1X)<br>431 (1X)	233 (1X)<br>275 (2X)<br>896 (1X)	-8 (2X)<br>275 (2X)
A:T → C:G	ND	233 (1X)	104 (1X)<br>275 (1X)
A:T → T:A	401 (1X)	ND	109 (2X)<br>233 (1X)<br>473 (1X)
C:G → T:A	ND	29 (2X)<br>632 (1X)	1034 (2X)
C:G → A:T	ND	808 (1X)	ND
C:G → G:C	67 (1X)	ND	ND
ME insertion	-34 (1X)	ND	ND
