# Supplementary material for: Role of error-prone DNA polymerases in spontaneous mutagenesis in Caulobacter crescentus
Source: Genet Mol Biol. 2020 Mar 9;43(1):e20180283. doi: 10.1590/1678-4685-GMB-2018-0283 (PMC7198004; doi:10.1590/1678-4685-GMB-2018-0283)
Supplement: Supplementary file 4 [file 1415-4757-GMB-43-1-e20180283-s1.pdf]

Genetics and Molecular Biology, 43, 1, (2020)

Supplementary Material to "Role of error-prone DNA polymerases in spontaneous mutagenesis in *Caulobacter crescentus*"

C insertion

GCCTGGCCGGCAGCGCGCCGTTCCGCATCGG CCTGCTCTACGACAACCCCTCGACGGGCTA

Figure S1 - Mutational hotspot in the *xylR* coding region. A small region of the *xylR* gene is shown, from bases 200 to 260 of the ORF. Insertion of cytosine after base 230, as indicated by the arrow, is the predominant type of mutation in this gene. Only the coding strand and the corresponding insertion is shown for simplicity.
